# Retraction Note: Determinants of suboptimal breastfeeding practice in Debre Berhan town, Ethiopia: a cross sectional study

**DOI:** 10.1186/s13006-018-0155-z

**Published:** 2018-03-07

**Authors:** Teklemariam Gultie, Girum Sebsibie

**Affiliations:** 1 Department of Midwifery, College of Medicine and Health Science, Arba Minch University, Arba Minch, Ethiopia; 2 Department of Nursing and Midwifery, College of Health Science, Addis Ababa University, Addis Ababa, Ethiopia

## Retraction note

The authors are retracting this article [1] because there is significant overlap of both text and data with the Master’s Thesis of Hilina Ketma, “Assessment of prevalence and determinants of suboptimal breast feeding among mothers of children aged less than two years in Dire Dawa City Administration, Ethiopia, June 2013”, which was defended at the School of Graduate Studies, Addis Ababa University, in June 2013.
